# Reversible Structure Engineering of Bioinspired Anisotropic Surface for Droplet Recognition and Transportation

**DOI:** 10.1002/advs.202001650

**Published:** 2020-07-26

**Authors:** Qian Li, Lijun Li, Kui Shi, Baisong Yang, Xin Wang, Zhekun Shi, Di Tan, Fandong Meng, Quan Liu, Shiqi Hu, Yifeng Lei, Sheng Liu, Longjian Xue

**Affiliations:** ^1^ School of Power and Mechanical Engineering, The Institute of Technological Science Wuhan University South Donghu Road 8 Wuhan 430072 China

**Keywords:** bioinspiration, directional wetting, droplet recognition and transportation, pillar arrays, smart surfaces

## Abstract

Surfaces with tunable liquid adhesion have aroused great attention in past years. However, it remains challenging to endow a surface with the capability of droplet recognition and transportation. Here, a bioinspired surface, termed as TMAS, is presented that is inspired by isotropic lotus leaves and anisotropic butterfly wings. The surface is prepared by simply growing a triangular micropillar array on the pre‐stretched thin poly(dimethylsiloxane) (PDMS) film. The regulation of mechanical stress in the PDMS film allows the fine tuning of structural parameters of the micropillar array reversibly, which results in the instantaneous, in situ switching between isotropic and various degrees of anisotropic droplet adhesions, and between strong adhesion and directional sliding of water droplets. TMAS can thus be used for robust droplet transportation and recognition of acids, bases, and their pH strengths. The results here could inspire the design of robust sensor techniques.

## Graphical abstract

The dynamic tuning of the wetting state of a droplet on a surface is realized by an asymmetric surface microstructure and mechanical stress. The proposed surface, TMAS, can switch in situ between isotropic and various degrees of anisotropic adhesions to water droplets, and can be used for the robust transportation of droplets and the recognition of acids, bases, and their pH strengths. TMAS shows the capability of in situ reversibility and fast response, demonstrating the future applications for sensor techniques.

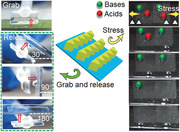

## 1 Introduction

Surfaces with micro‐ and nanoscale features provide key functions for many plants and insects to survive in nature.^[^ ^1^ ^]^ For instance, anisotropic surfaces enable butterflies to get rid of water droplets along the outward direction of wings,^[^ ^2^ ^]^ water striders to walk on water,^[^ ^3^ ^]^ plants to trap pollen and insects.^[^ ^4^ ^]^ The ordered, direction‐dependent geometrical features result in anisotropic wettability and water droplet adhesions. By mimicking natural surfaces, diverse engineered materials with anisotropic wetting properties have made contribution to advanced applications in intelligent microfluidic, printing industry, self‐cleaning coating, biomedical applications, and so on.^[^ ^5^ ^]^ For instance, Li et al.^[^ ^5g^ ^]^ developed a pitcher‐plant‐inspired topological fluid diode that allows a rapid, directional, and long‐distance transport of liquid. However, the constant isotropic/anisotropic wetting properties due to the stationary surface structure/component significantly limit their applications in the emerging area of intelligent techniques and devices.

The surfaces with the ability to reversibly switch between distinct wetting states have attracted enormous research interests,^[^ ^6^ ^]^ which holds great potential for the utilizations, such as rewritable devices,^[^ ^7^ ^]^ wearable materials,^[^ ^8^ ^]^ cell capture.^[^ ^6d^ ^]^ As the wettability of a substrate is mainly determined by the surface energy and the morphology (roughness) of the surface, smart materials responsible to external stimulus, like magnetic field, electric field, temperature, light, pH value, etc. have been used to alter the surface chemistry and/or topography, and therefore the wetting states.^[^ ^9^ ^]^ Wang et al.^[^ ^9a^ ^]^ proposed an electrochemical strategy to reversibly change the surface component, enabling the in situ reversible switch between underwater superoleophilicity and superoleophobicity. Dynamic surface microstructure has been widely demonstrated to be an effective way to realize the switch between Cassie and Wenzel states, which usually allows the transition from slippery to sticky state of droplets.^[^ ^8^, ^10^ ^]^ Among these techniques, mechanical configuration shows a high feasibility for the instant wettability tailoring.^[^ ^10e,f^ ^]^ For instance, Wang et al.^[^ ^8^ ^]^ reported a finger print‐mimicking elastic surface that can switch between the states of lotus leaf and rose petal by in‐plane lateral strains. However, the dynamic changes of surface properties usually need to be carried out ex situ, which inhibits their potential applications.^[^ ^6^, ^9^, ^10^ ^]^ On the other hand, no report has demonstrated the in situ dynamic, reversible switching between the isotropic (i.e., lotus‐leaf‐like) and various degrees of directional wetting (i.e., butterfly‐wing‐like) states. Moreover, while those smart surfaces can be used to transport droplets, the ability to analyze the property of droplets and to sort them is still missing.

Herein, we present a smart surface possessing the ability to reversibly and in situ manipulate water droplet and to distinguish acidic/basic liquids, and their strengths by mechanical strain. The surface is prepared by growing triangular micropillar array on a pre‐stretched thin poly(dimethylsiloxane) (PDMS) film, which is referred to as TMAS throughout the context. The mechanical stretching and relaxation of TMAS allow the triangular micropillar array to possess dynamic periodic parameters. It thus changes the contact lines at advancing and receding fronts of a droplet on the TMAS, enabling the in situ switching between isotropic and directional wetting states. TMAS can also be reversibly switched between strong droplet‐adhesion and droplet‐sliding states by mechanical stretching or rotating. Therefore, TMAS possesses the abilities to transport water droplets and to identify acidic and basic liquids and their pH strengths. We believe the work here not only provides a smart surface for droplet manipulation, but may also initiate new sensor techniques.

## 2 Results and Discussion

### 2.1 Fabrication of TMAS

**Figure 1:**
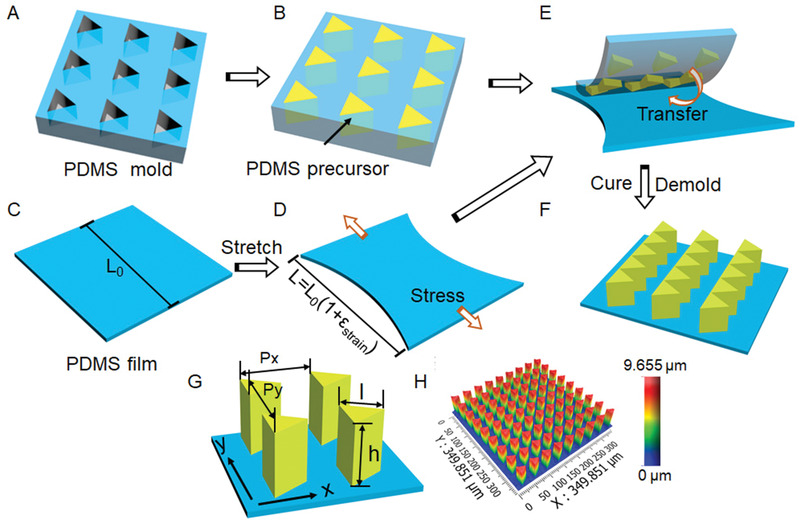
Schematic illustration of the fabrication of TMAS. A) A PDMS mold with an array of triangular holes. B) PDMS precursor was spread on PDMS mold and the excess PDMS precursor was removed. C–D) PDMS film with thickness of 0.6 mm was stretched in a designed direction (*x* or *y* direction) to a defined strain, *ε*_strain_. E) PDMS precursor‐filled mold was brought into contact with the stretched PDMS film and cured at 90 °C for 1 h. F) TMAS was ready after releasing from the mold. G) The definition of pillar height (*h*), side length of triangular tip (*l*), the periods of *P_x_* and *P_y_*, directions of *x* and *y*, respectively. H) A typical 3D image of TMAS with *ε*_strain_ = 0.

TMAS, which is composed of PDMS backing layer and triangular micropillar array, was successfully fabricated by soft lithography (**Figure** **1** and Figure S1, Supporting Information). Briefly, PDMS precursor was filled into the triangular microholes in a PDMS mold, whose height, side length and period are 10, 25, and 40 µm, respectively (Figure 1A,B). The PDMS precursor‐filled mold was then brought into contact with a pre‐stretched PDMS film (Figure 1C–E), followed by a proper curing at 90 °C for 1 h (Figure 1E).^[^ ^11^ ^]^ After peeling off from the mold and the relaxation of stress in PDMS backing layer, TMAS was ready (Figure 1F). When the pre‐stretching of PDMS film is parallel to the direction of triangle corner pointing, it is defined as the x direction (Figure 1G), while the orthogonal direction of pre‐stretching is defined as the *y* direction. The corresponding periodic distances in the *x* and *y* directions are then noted as *P_x_* and *P_y_*, respectively. Depending on the pre‐strain *ε*_strain_ = ((*L* − *L*_0_)/*L*_0_) × 100%, where *L*_0_ and *L* are the lengths of PDMS film before and after stretching, and the direction of pre‐stretching, the resulted samples are referred as TMAS*‐xε*_strain_ or TMAS*‐yε*_strain_, like TMAS*‐x*40% or TMAS*‐y*40%, in the following text. Without pre‐stretching of PDMS film (0% strain), the resulted TMAS faithfully copied the parameters of PDMS mold, showing period, height and side length of 40, 10, and 25 µm, respectively (Figure 1H).

**Figure 2:**
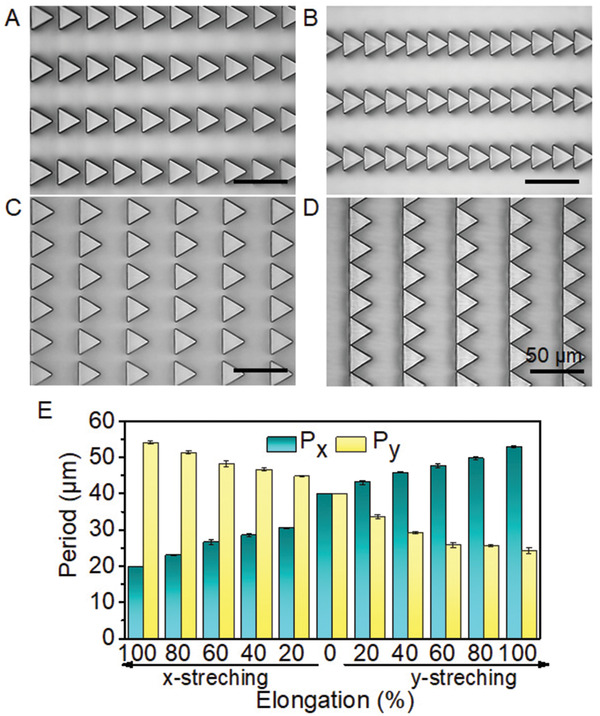
Surface topography of TMAS. A–D) Typical optical image of TMAS prepared with pre‐stretching in the *x* direction at a strain of 40% (A) and 100% (B); and with pre‐stretching in the *y* direction at a strain of 40% (C) and 100% (D). The resulted periods in the *x* and *y* directions at various stretching were summarized in (E). Each data point in (E) represents the mean value of three measurements. Standard deviations are indicated by error bars.

Depending on the direction and *ε*_strain_ of pre‐stretching, various periodic parameters of TMAS were achieved from the single template (**Figure** **2** and Figure S2, Supporting Information). When the PDMS film was pre‐stretched in the *x* direction, the relaxation of strain shortened *P_x_* and enlarged *P_y_* (Figure 2A and Figure S2A–C, Supporting Information). Once *ε*_strain_ reached 100% in the *x* direction, the resulted TMAS presented a “head‐to‐tail” arrangement in the *x* direction (Figure 2B). In contrast, the pre‐strain in the *y* direction shortened *P_y_* and enlarged *P_x_* (Figure 2C and Figure S2D,F, Supporting Information). The *y* 100% strain yielded a “shoulder‐by‐shoulder” arrangement of pillars (Figure 2D). Therefore, the systematic change of pre‐stretching in the *x* and *y* directions allowed us to continuously tune *P_y_* from 54.1 ± 0.4 to 24.2 ± 0.7 µm with *P_x_* changing from 19.9 ± 0.1 to 53.0 ± 0.3 µm accordingly (Figure 2E). For comparison, the surfaces with circular (Figure S3A–D, Supporting Information), squared (Figure S3E–H, Supporting Information) and hexagonal (Figure S3I–L, Supporting Information) micropillar arrays were also prepared via the same technique. Similarly, systematically changed *P_x_* and *P_y_* (Figure S3D,H,L, Supporting Information) and the “shoulder‐by‐shoulder” arrangement of pillars (Figure S3C,G,K, Supporting Information) were realized. It is worth mentioning that only one template was used for each micropillar geometry, though a series of periodic parameters (*P_x_* and *P_y_*) were achieved.

### 2.2 Controllable Adhesion of Water Droplets

**Figure 3:**
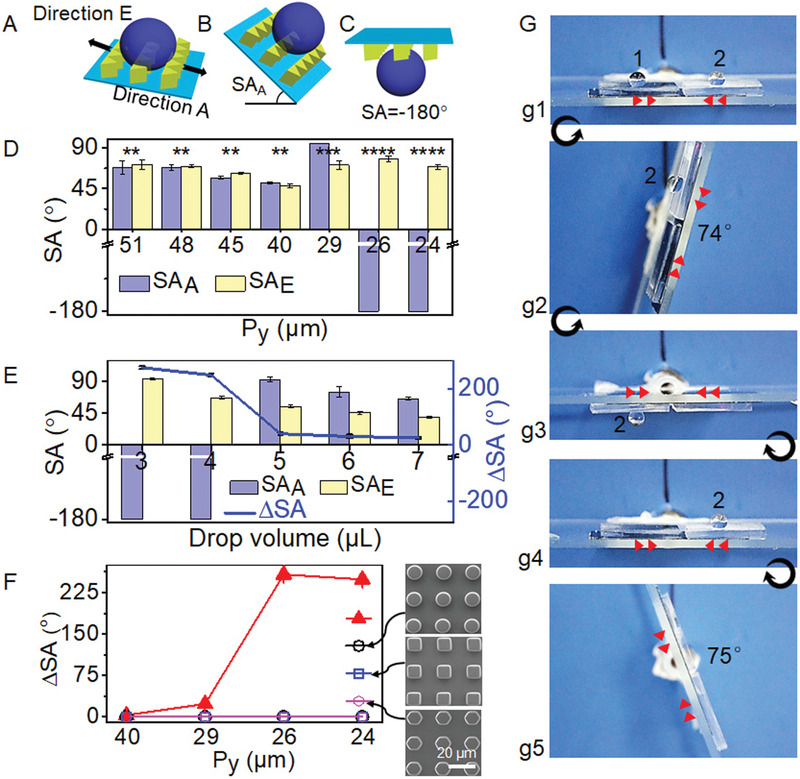
Demonstration of anisotropic water adhesion on TMAS. A,B) Definition of direction *A* and *E*, sliding angle (SA*_A_* and SA*_E_*) of a water droplet on TMAS. C) If a droplet sticks on TMAS with upside down, SA is defined as −180°. D) Dependence of SA*_A_* and SA*_E_* on the period of triangular pillars in the *y* direction (*P_y_*). The volume of water droplet was 4 µL. *N* = 3, mean ± s.d., one‐way analysis of variance (ANOVA) test, **p > 0.05, ***p < 0.001, ****p < 0.0001. E) Dependences of SA*_A_*, SA*_E_* and ΔSA = |SA*_A_* − SA*_E_*| on the water droplet volume on TMAS*‐y*100%. F) Dependence of ΔSA on *P_y_* of triangular, circular, square, and hexagonal micropillars arrays. G) Snapshots showing the direction‐dependent droplet adhesion on TMAS*‐y*100%. The red arrow heads indicate the direction of the triangular pillars on TMAS, the black arrows between snapshots indicate the rotating direction. Each data point in (D–F) represents the mean value of three measurements. Standard deviations are indicated by error bars.

The ability of water adhesion on TMAS was assessed by the sliding behavior of water droplet.^[^ ^12^ ^]^ The sliding direction of water droplet towards the angle of the triangle micropillar is defined as direction *A*, the opposite direction towards the bottom edge of the triangle micropillar is defined as direction *E* (**Figure** **3**A). When the droplet slides along direction *A* or *E*, the corresponding sliding angle (SA) is referred as SA*_A_* (Figure 3B) or SA*_E_*, respectively. If the droplet cannot slide on the surface, the SA is defined as −180° in order to distinguish from the case that the water droplet slides at an angle infinitely close to 180° (Figure 3C). The difference in SAs in opposite directions, ΔSA = |SA*_A_* − SA*_E_*| is then used to define the surface to be isotropic and anisotropic. When the PDMS film was not pre‐stretched at any direction (*P_x_* = *P_y_* = 40 µm), the water droplets could slide in both directions with SA*_A_* = 51.3 ± 1.4° and SA*_E_* = 48.1 ± 1.8° (Figure 3D). The negligible difference of SAs in opposite directions along *x* axis (ΔSA = 3°) and the identical SAs in *y* axis (Figure S4A, Supporting Information) allow us to consider this surface to be macroscopically isotropic, though the microstructure is anisotropic in the orientation of *x* axis.

The period and structure of pillars determine the adhesion of water droplets. On all the TMASs with *P_y_* larger than 40 µm, water droplets can slide in both directions of A and E. When *P_y_* increased from 40 to 51.6 µm, SA_A_ increased from 51.3 ± 1.2° to 67.9 ± 7.7° and SA*_E_* increased from 48.1 ± 1.8° to 71.3 ± 1.4° (Figure 3D). As there is no statistic difference in SAs in directions *A* and *E*, TMASs with *P_y_* ≥ 40 µm are thus considered as isotropic for water adhesion. On the other hand, when *P_y_* decreased from 40 to 29.2 µm, SA*_A_* sharply increased to 95° while SA*_E_* also increased to 70.8 ± 1.4°, showing a statistic difference in opposite directions with ΔSA = 23.5 ± 2.4°. Upon the further decrease in *P_y_*, that is, *P_y_* = 25.8 and 24.3 µm, water droplet could not slide in direction *A*, even with the surface turned upside down, showing a SA*_A_* of −180°. In the opposite direction, however, water droplets can still slide away with SA*_E_* around 70°. With a ΔSA ranging from 23.5 ± 2.4° to ≈250°, these surfaces possess the ability of directional adhesion (anisotropic). Moreover, the anisotropy (ΔSA) of TMAS, taking of TMAS*‐y*100% as the example, strongly depends on the volume of water droplet (*V*) (Figure 3E). Upon the increasing of *V* from 3 to 7 µL, all the droplets slid away from the surface in direction *E* and SA*_E_* decreased from 94.2° to 39.1°. In direction *A*, water droplet with *V* ≤ 4 µL stuck on the surface, while SA*_A_* showed the same tendency as SA*_E_* with *V* ≥ 5 µL. That is, TMAS shows strong anisotropic adhesion (ΔSA ≈274.9 ± 5.0°) to water droplet with *V* ≤ 4 µL, while the anisotropy is much weaker (ΔSA ≈ 25.2 ± 4.3°) when *V* ≥ 5 µL. It is reasonable that a large droplet provides a large component of gravity along the tilted surface which could initiate the rolling of droplet in any direction. Therefore, it suggests the possibility to finely tune the anisotropy of TMAS by regulating the *P_y_* (Figure S4A, Supporting Information). In addition to the droplet size, the size of the triangular array (such as vertex angle or base length) also has an effect on the surface anisotropy.^[^ ^13^ ^]^ On the other hand, however, there is no difference in the SA between directions of +*y* and −*y* (Figure S4B, Supporting Information). Moreover, the arrays of pillars with circular, squared, and hexagonal shapes showed isotropic behaviors in both *x* and *y* directions, regardless of the drop volume (taking 4 and 6 µL as the examples) (Figure S5, Supporting Information). It clearly indicates the importance of the geometry design for TMAS and the ability to switch between isotropic and directional adhesion (Figure 3F). TMAS can be switched from the state of isotropic adhesion to water droplets (ΔSA = 0°) to the state of strongly anisotropic in opposite directions (along the *x* axis) (ΔSA = 258.3 ± 2.5°) by simply regulating the *P_y_*. It also suggests the existence of a critical value of *P_y_* in TMAS to switch TMAS between isotropic and directional adhesion to water droplets. With the specific size of the triangular micropillars here, the critical value is around 29 µm. In contrast, however, the surfaces composed of circular, squared and hexagonal micropillar arrays remain isotropic, no matter the *P_y_* values.

**Figure 4:**
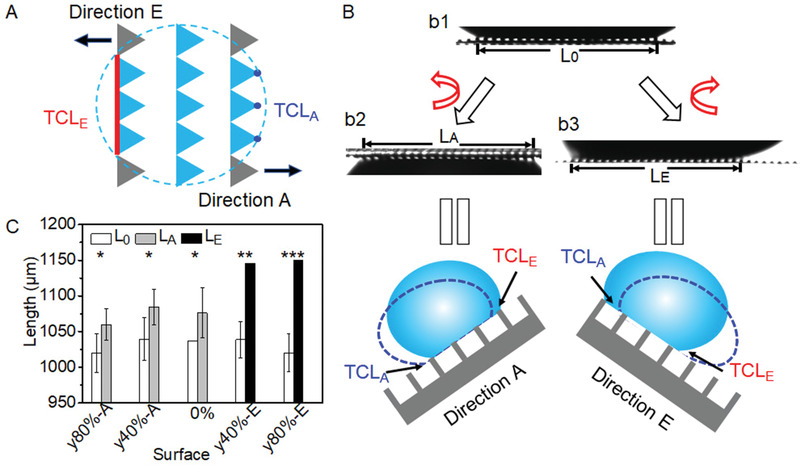
Mechanism of the directional droplet adhesion. A) Schematic illustration of three‐phase contact line (TCL) at the edges (TCL*_E_*) and the angles (TCL*_A_*) of triangular pillars on TMAS. The dash circle indicates the contact perimeter of a droplet. B) Contact interface of a water droplet on (b1) horizontal, (b2) direction‐*A*, and (b3) direction‐*E* rotated TMAS. Contact length between the advancing and receding fronts at initial state (*L*_0_) and that before the droplet sliding at tilted state in direction *A* (L*_A_*) and *E* (L*_E_*) are indicated. The locations of corresponding TCLs are indicated in the schematic drawing. C) Change of L*_A_* and L*_E_* on TMAS after the rotation in direction *A* and *E*. *N* = 3, mean ± s.d., one‐way analysis of variance (ANOVA) test, *p > 0.05, **p < 0.05, ***p < 0.01. Each data point represents the mean value of three measurements. Standard deviations are indicated by error bars.

**Figure 5:**
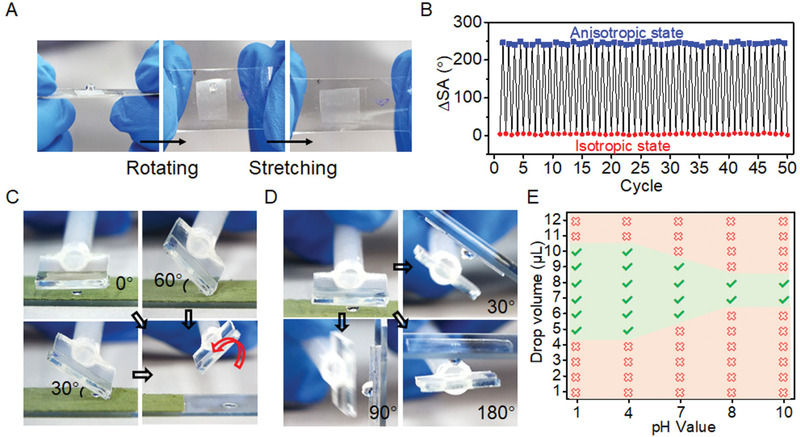
Two modes for controllable droplet manipulation by TMAS. A) Dynamic turning off the droplet adhesion by stretching the TMAS at a tilted state. B) Cyclic switching between isotropic (ΔSA of ≈5°) and anisotropic (ΔSA of 247°) droplet adhesion by mechanical strain and relaxation. C) The picking up of a droplet from various approaching angles and the releasing of droplet by rotating TMAS in direction *E* (red arrow). D) The easy releasing of droplet onto tilted, vertical and upside‐down surfaces by simply rotating TMAS in direction *A*. E) The transferable volume of a droplet with different pH values by TMAS*‐y*100%. A cross (hook) means the droplet can't (can) be transferred.

The directional adhesion of water droplet was further demonstrated by a macroscopic test on a homemade rotating stage (Figure 3G and Movie S1, Supporting Information). Two pieces of TMAS*‐y*100% were mounted on the stage with the left one having its direction *E* pointing left and the right one has the opposite direction. Each sample was placed with one water droplet of 4 µL (Figure 3G, 1). Upon the anti‐clockwise rotating of the stage, the droplet on the left sample stuck on the surface until a critical tilting angle of 74° was reached (Figures 3G, 2). In contrast, the droplet on the right sample kept sticking on the surface even the surface was upside down (Figure 3G). The followed backwards rotating dropped the droplet at the angle of 75° (Figures 3G, **4**–**5**). It clearly confirmed the adhesion anisotropy of water droplet on the surface.

### 2.3 Mechanism of the Smart Droplet Adhesion

In order to understand the mechanism for the directional adhesion of water droplet, the liquid‐solid interface was examined in detail. When a water droplet was placed on TMAS, it sat on top of the micropillar array but did not penetrate into the space among pillars, indicating a contact mode of Cassie (Figure S6A, Supporting Information).^[^ ^9^, ^10^ ^]^ Because of the geometry of the triangular pillars, there are two different contact geometries at the three‐phase contact line (TCL) perpendicular to the *x* direction (**Figure** **4**A). Taking TMAS*‐y*100% as an example, a long TCL forms along the edge of triangular pillars (TCL*_E_*, red line), while discrete contact points form at the corner of triangular pillars (TCL*_A_*, blue dots). When a water droplet slides, the energy barrier at TCL*_E_* is much larger than that at TCL*_A_* (Figure S6B, Supporting Information),^[^ ^14^ ^]^ which is similar to the friction behavior of the array of triangular micropillars on a solid surface.^[^ ^15^ ^]^ Therefore, TCL*_E_* pins the TCL of a droplet in place. The pinning effect of TCL*_E_* reaches its maximum on the geometry of “shoulder‐by‐shoulder” and gets smaller when *P_y_* increases. When *P_y_* is larger than ≈29 µm, the pinning effects of TCL*_E_* and TCL*_A_* get close that the surface turns into isotropic for water adhesion.

The sliding behavior of a water droplet (4 µL) in the directions of *A* and *E* on TMAS was investigated by monitoring the distance between the advancing and receding fronts (*L*); that is, the distance between TCL*_E_* and TCL*_A_* (Figure 4B).^[^ ^8^, ^9^ ^]^ At the horizontal state, the water droplet on the surface was symmetric, showing an initial distance of *L*_0_ (Figures 4B,  1). When the surface was tilted towards the direction *A* (Figures 4B,  2), TCL*_A_* and TCL*_E_* of water droplet were at the advancing and receding front, correspondingly. Because of the strong pinning effect of TCL*_E_*, the receding front of the droplet pinned in‐place. (In few cases, TCL*_E_* moved to the next row of pillars, which might be caused by the defects in TCL*_E_*.) The further tilting of TMAS caused the forward moving of advancing front slightly. The forward moving of the advancing front made the droplet flattened on the surface (sticking better). Meanwhile, the surface tension of water droplet prevents it from further flattening. Thus, on the surfaces of TMAS*‐yε*_strain_ (*ε*_strain_ ≥40%), the droplet kept adhering to the surface, even the surface was turned upside‐down. It is worth mentioning here that the droplet on TMAS is in the Cassie mode even the water droplet sticks firmly to the surface. Therefore, a simple rotating can shift TMAS between the states of super‐sticky and easy‐sliding of water droplet. It is quite different from the previous reports that the strong adhesion of a droplet on the surface mainly originates from the Wenzel mode, which makes these surfaces can hardly change from the strong sticky to the easy sliding state, not to mention in situ.^[^ ^8^, ^10^ ^]^ Statistically, there was no significant difference between *L*_A_ and *L*_0_ (Figure 4C), which confirms the sticking of water droplet on the surface.

When TMAS was tilled towards the direction *E*, TCL*_A_* and TCL*_E_* of the water droplet shifted their positions that TCL*_E_* was located at the advancing front and the receding front was TCL*_A_*. When a critical tilting angle is reached, the receding front passes across the pillar top quickly and jumps to the next row of pillars, rather than stopping at the base of the triangle on the same pillar.^[^ ^16^ ^]^ Therefore, the receding front slid downhill while the advancing front was pinned, which resulted in a higher distortion of the droplet (Figures 4B, 3). The further inclination of substrate increased the distortion of the droplet. The mass center of droplet shifted downhill, causing the droplet surface to descend onto the next micropillars without moving the contact line.^[^ ^16^ ^]^ Macroscopically, however, it looks like that the advancing front moved forward. The forward‐moving distance was larger than that of the sliding distance of receding front, resulting in a significant increase in *L* (*L*_E_) (Figure 4C). Therefore, the further tilting of the substrate forced the droplet to continuously descend onto the next pillars, resulting in the rolling off of the droplet.

### 2.4 Mechanical Regulation of Water Adhesion

Taking advantage of the mechanical switching between states of isotropic and directional adhesion, in situ regulation of water adhesion was then demonstrated with TMAS*‐y*100% (**Figure** **5**). TMAS*‐y*100% has a ΔSA of 247° at the stress‐released state and shifts to the isotropic state (ΔSA of ≈5°) upon the stretching in the *y* direction. Therefore, the adhered droplet on TMAS*‐y*100% can slide away from the surface upon the rotating and stretching (Figure 5A and Movie S2, Supporting Information). The switching is totally reversible that no obvious change in ΔSA was found after 50 cycles of mechanical stretching and relaxation (Figure 5B). TMAS also showed powerful ability to transport water droplets. TMAS could pick up a droplet from various tilting angles and be ready to release the droplet by just rotating TMAS in the direction *E* (Figure 5C and Movie S3, Supporting Information). Depending on the stretching state, or in other words the value of *P_y_*, TMAS*‐y*100% can transfer droplets with various volumes (from 2 to 12 µL). The maximum droplet volume which TMAS can transport decreases when *P_y_* increases (Figure S7A, Supporting Information). For instance, TMAS with *P_y_* of 40 µm can transfer droplet with volume up to 12 µL, while only the droplet with volume no more than 8 µL can be transferred by TMAS with *P_y_* of 24 µm. Depending on the *P_y_* value and the area of TMAS, it can therefore transport several droplets with various volumes at the same time (Figure S7B and Movie S4, Supporting Information). Besides transporting to a horizontal surface, TMAS can pick up a droplet and deposit it onto tilted, vertical, and even upside‐down surfaces (Figure 5D and Movie S5, Supporting Information), taking advantage of the strong adhesion in direction A. Moreover, TMAS can also transport water droplet with a wide range of pH values (pH = 1–10) (Figure 5E). Acidic droplets (pH = 1, 4) with volume ranging from 5 to 10 µL can be easily transported by TMAS*‐y*100%, while a narrow volume range between 7 and 8 µL of alkaline droplets (pH = 8, 10) could be handled.

**Figure 6:**
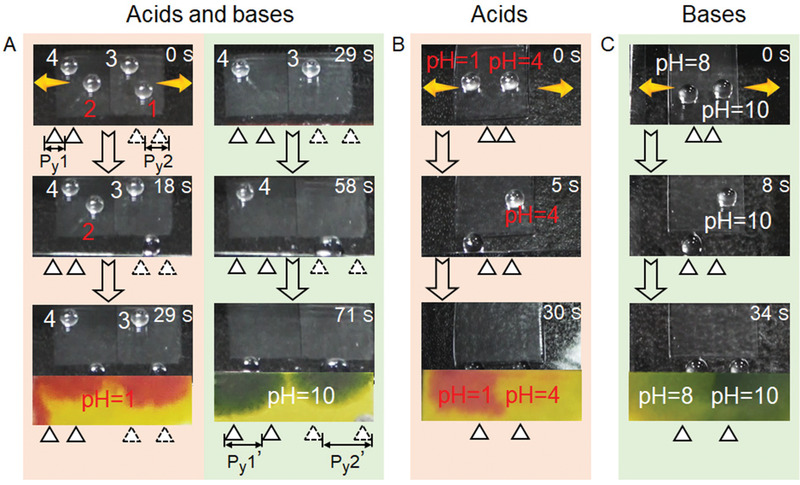
The ability of TMAS to distinguish acids, bases and acid/base strength. A) Snapshots showing the recognition of acids and bases on TMAS*‐y*100% (left) and TMAS*‐y*80% (right). Droplets 1 and 2 have pH = 1. Droplets 3 and 4 have pH = 10. B,C) Snapshots showing the identification of acid/base strength. The orange arrows indicate the stretching direction. The schematic below the image indicates the orientation and *P_y_* of triangular pillars on the corresponding TMAS. In the schematic, the size of the triangles (the top of triangular pillars) and the space between triangles are not to scale. The time on the top right corner represents the moment when the droplet slides.

TMAS also possesses the exciting ability to distinguish acids and bases and to tell pH values by mechanical stress (**Figure** **6**). To demonstrate this ability, TMAS*‐y*100% (left) and TMAS*‐y*80% (right) were welded together as the demo (Figure 6A). One acidic droplet (HCl, pH = 1, *V* = 4 µL, droplet 1 and 2) and one alkaline droplet (NaOH, pH = 10, *V* = 4 µL droplet 3 and 4) were placed on each TMAS. By slowly stretching the TMAS in the *y* direction, the acidic droplet 1 falls first (18 s), and then the acidic droplet 2 (29 s); the further stretching released alkaline droplet 3 and 4 successively (58 and 71 s) (Movie S6, Supporting Information). It is quite clear that TMAS is able to identify acid and base solutions via simple mechanical stress (instantaneous adjustment of structural parameters), even the droplets have an identical appearance. The position switching of the acidic and alkaline droplets on TMAS will not change the sliding sequence (Figure S8 and Movie S7, Supporting Information), which confirms the ability of recognition of acidic and alkaline liquids. It has been reported, the presence of HCl (NaOH) decreases (increases) the surface tension of the solution comparing with water.^[^ ^17^ ^]^ A lower surface tension of the solution would facilitate the distortion of the droplet during the tilting of TMAS (Figure 3B), making the droplet easier to roll off. Hence, it is easier for an acidic droplet to slide than a basic droplet. It infers that there could be a particular *P_y_* of TMAS allowing the droplet with corresponding surface tension to slide. In another word, the value of *P_y_* can distinguish the droplets with same volume as acid or base.

In addition to the recognition of acid and base liquids, TMAS can also identify the strength of acid/base. As the concentration of HCl (NaOH) increases, the surface tension of the solution decreases (increases) comparing with water.^[^ ^17^ ^]^ The stronger the acidity is, the easier for an acidic droplet to move; and the stronger the basicity is, the harder for a basic droplet to move from the TMAS surface (Figure 6B,C, Movie S8 and S9, Supporting Information). Meanwhile, TMAS shows high resistance to acids and bases. The immersion in HCl solution (pH = 3, 4, 5) or NaOH solution (pH = 8, 9, 10) for 7 days at room temperature has no detectable influence on the SAs in all the directions (and thus ΔSAs) (Figure S9, Supporting Information), that the function to recognize liquid acidity/basicity stayed the same. Moreover, the switching ability between isotropic and directional adhesion also remained steady (Figure S10, Supporting Information). The high stability and reversibility of TMAS thus guarantee a high potential to serve as a pH sensor.

## 3 Conclusions

We have presented a smart surface, TMAS, which can switch reversibly between states of isotropic and directional water adhesions and possesses the ability to recognize the acidity/basicity of a liquid. TMAS was prepared by growing triangular micropillar array on the pre‐stretched thin PDMS film. The array of triangular micropillars offers a long TCL*_E_* in direction *A* and discrete TCL*_A_* in direction *E* at the receding front of a droplet on TMAS, which endows TMAS with directional adhesion to water droplet. Therefore, the adhesion of a water droplet on TMAS can be regulated by the opposite rotating. The mechanical stretching‐releasing cycle allows the easy, reversible regulation of TCLs at the advancing and receding fronts of a droplet and ultimately allows the transition between isotropic (lotus‐leaf‐like) and directional adhesion (butterfly‐wing‐like) of water droplets. TMAS can therefore be used to conveniently pick up and transfer droplet to horizontal, tilted, vertical, and even upside‐down surfaces. Moreover, TMAS has the ability to distinguish acids and bases and their pH strengths. The results here not only provide a new smart surface for droplet manipulation and identification, but also offer a new design principle for new chemical sensors, and so on.

## 4 Experimental Section

##### Materials and Preparation

PDMS elastomer kit (Sylgard 184) was purchased from Dow Corning (MI, USA). PDMS precursor was prepared by properly mixing the prepolymer and cross‐linker in a ratio of 10:1 (by weight), followed by degassing in a desiccator for 0.5 h. The fabrication of TMAS can be roughly considered as two steps: (1) PDMS films were prepared by filling PDMS precursor into a 0.6 mm thick chamber, which consists of two glass slides sandwiched with a 0.6 mm‐thick spacer, followed by curing at 90 °C for 1 h. The fully cured PDMS films were peeled off from the mold and cut into a rectangular shape (35 × 15 mm^2^). The resulted PDMS films were clamped on a glass sheet and uniaxially stretched to the predefined degrees. (2) PDMS precursor was poured over the triangular pillar arrays on silicon substrate which was prepared by standard photolithography. After degassing for ≈3 min in vacuum, the prepolymer was cured at 90 °C for 1 h. PDMS negative mold can be obtained after peeling off carefully from the silicon substrate. The PDMS molds were coated with 1H,1H,2H,2H perfluorodecyltriethoxysilane following the procedure in a previous study.^[^ ^11^ ^]^ The triangular micropillar arrays were prepared by pouring PDMS precursor onto the PDMS mold (excess PDMS precursor on the mold was carefully scraped off). The precursor‐filled PDMS mold was then brought into contact with the pre‐stretched PDMS film and cured at 90 °C for 1 h. After peeling off from the PDMS mold and the releasing of strain, TMAS was ready for use.

##### Characterization

The optical microscopy (ECLIPSE Ci‐L, Nikon) and field emission scanning electron microscopy (FE‐SEM, SIGMA, Zeiss AG, Germany, and MIRA 3 LMH, Tescan AG, Czech Republic) were employed to examine the surface morphology. Inverted confocal microscope (Leica TCS SP8 SMD, HCX PLAPO 40× dry objective) with a resolution of about 0.25 and 1.0 µm in the horizontal and vertical direction to investigate the state of water droplet on TMAS. Droplet sliding angle were measured by a droplet shape analysis (OCA25, Dataphysics, Germany) at ambient temperature. The water droplet volumes for SA measurements were 3–7 µL. For SA measurements, the rate of stage rotating was 1.68° per second. The average values of SA were obtained by measuring the same samples at least three different positions.

##### Statistical Analysis

All values were expressed as the mean ± standard error of the mean (SEM) of individual samples. Samples were analyzed by using one‐way analysis of variance (ANOVA) test. In all cases, values of *p* < 0.05 were considered as statistically significant. Statistical analysis was performed using Microsoft Excel.

## Conflict of Interest

The authors declare no conflict of interest.

## Supporting information

Supporting Information

MovieS1

MovieS2

MovieS3

MovieS4

MovieS5

MovieS6

MovieS7

MovieS8

MovieS9
