# Supplementary material for: Reversible Structure Engineering of Bioinspired Anisotropic Surface for Droplet Recognition and Transportation
Source: Adv Sci (Weinh). 2020 Jul 26;7(18):2001650. doi: 10.1002/advs.202001650 (PMC7509748; doi:10.1002/advs.202001650)
Supplement: Supplementary file 1 — Supporting Information [file ADVS-7-2001650-s001.pdf]

Copyright WILEY-VCH Verlag GmbH & Co. KGaA, 69469 Weinheim, Germany, 2020.

ADVANCED SCIENCE
Open Access

Supporting Information

for *Adv. Sci.*, DOI: 10.1002/advs.202001650

Reversible Structure Engineering of Bioinspired Anisotropic Surface for Droplet Recognition and Transportation

*Qian Li, Lijun Li, Kui Shi, Baisong Yang, Xin Wang, Zhekun Shi, Di Tan, Fandong Meng, Quan Liu, Shiqi Hu, Yifeng Lei, Sheng Liu, and Longjian Xue**

Copyright WILEY-VCH Verlag GmbH & Co. KGaA, 69469 Weinheim, Germany, 2018.

Supporting Information

Reversible structure engineering of bioinspired anisotropic surface for droplet recognition and transportation

*Qian Li, Lijun Li, Kui Shi, Baisong Yang, Xin Wang, Zhekun Shi, Di Tan, Fandong Meng, Quan Liu, Shiqi Hu, Yifeng Lei, Sheng Liu, Longjian Xue**

Qian Li, Lijun Li, Kui Shi, Baisong Yang, Xin Wang, Zhekun Shi, Di Tan, Fandong Meng, Quan Liu, Shiqi Hu, Prof. Y. Lei, Prof. S. Liu, Prof. L. Xue
School of Power and Mechanical Engineering
The Institute of Technological Science
Wuhan University
South Donghu Road 8, 430072, Wuhan, China
E-mails: xuelongjian@whu.edu.cn

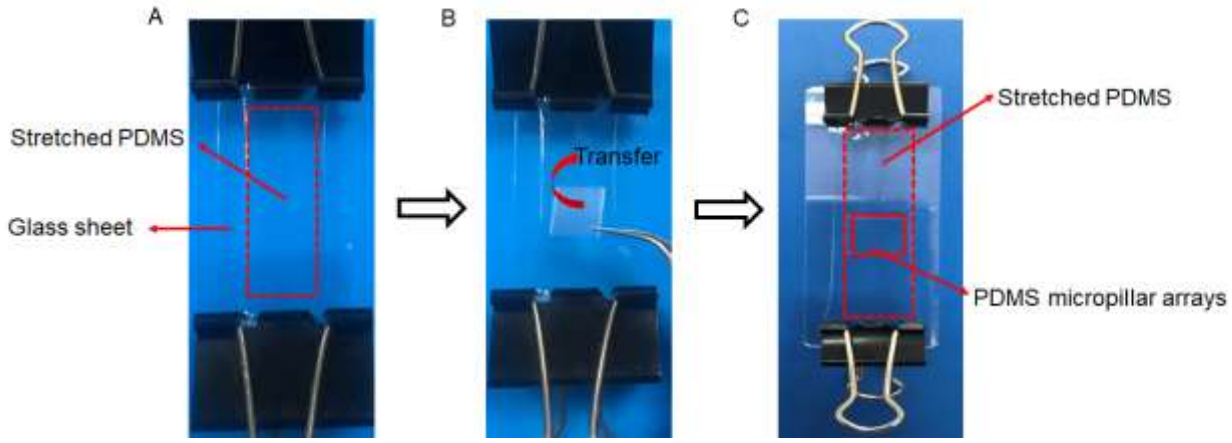

Figure S1. Fabrication process of TMAS. (A) Clamping PDMS film onto the glass slide by two cramps. The stretched PDMS is indicated by red dash line. (B) Putting upside-down PDMS mold, which was filled with PDMS precursor, onto the stretched PDMS; the assembly was then cured at 90 ℃ for 1 h. (C) TMAS (indicated by red solid line) was ready to peel off from the PDMS mold.

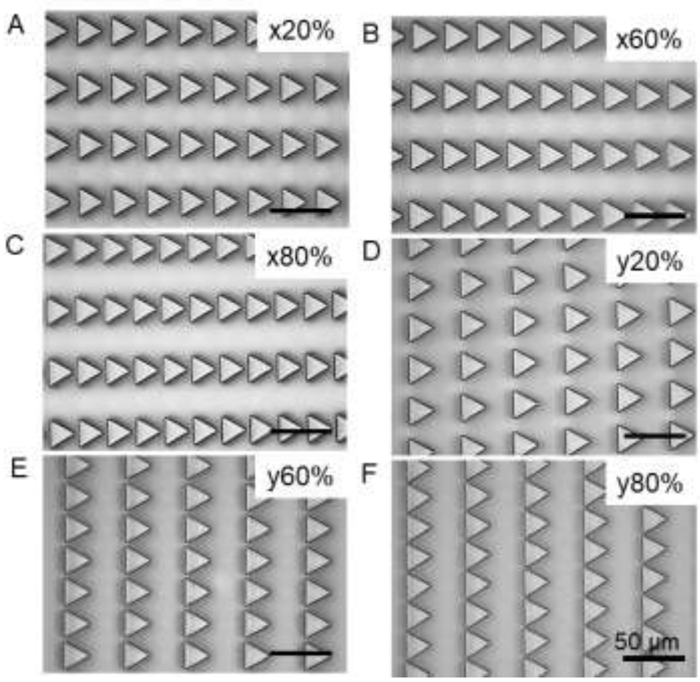

Figure S2. Surface topography of TMAS. Optical image of TMAS prepared with pre-strain in x direction at ε_{strain} of (A) 20%, (B) 60% and (C)80%; and with pre-strain in y direction at ε_{strain} of (D) 20%, (E) 60% and (F) 80%, respectively.

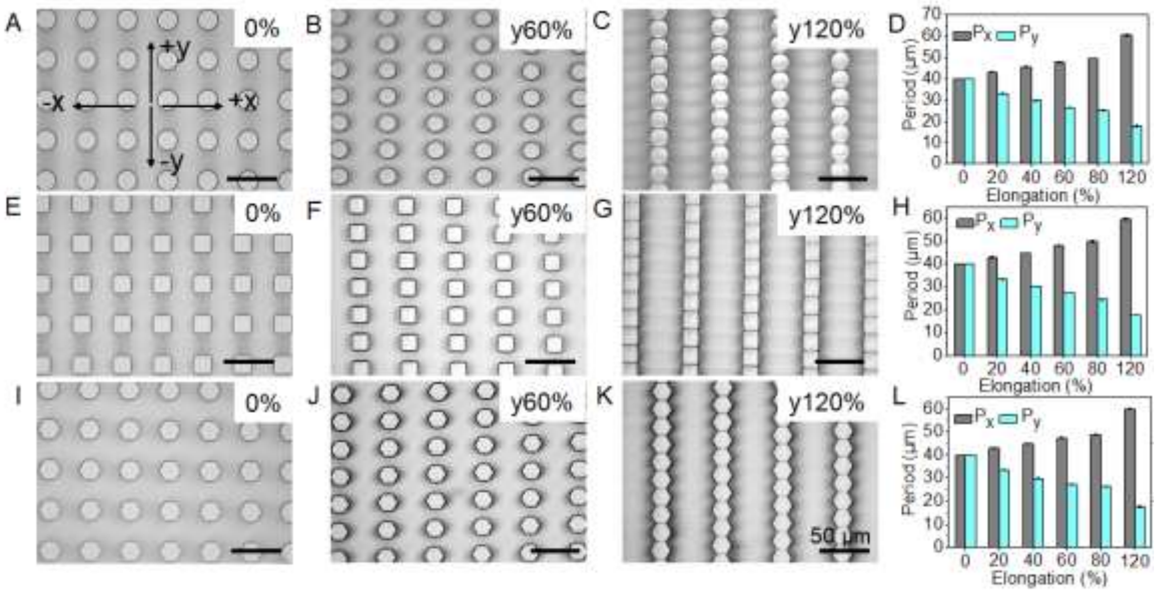

Figure S3. Surface topography of circular, squared and hexagonal micropillars array pre-stretched in y direction and the resulted periods. Optical image of circular, squared and hexagonal micropillars array prepared with pre-strain in y direction at a strain of (A, E, I) 0%, (B, F, J) 60% and (C, G, K) 120%. The resulted periods in directions y withvarious stretching were summarized in (D, H, L). Each data point represents the mean value of three measurements. Standard deviations are indicated by error bars.

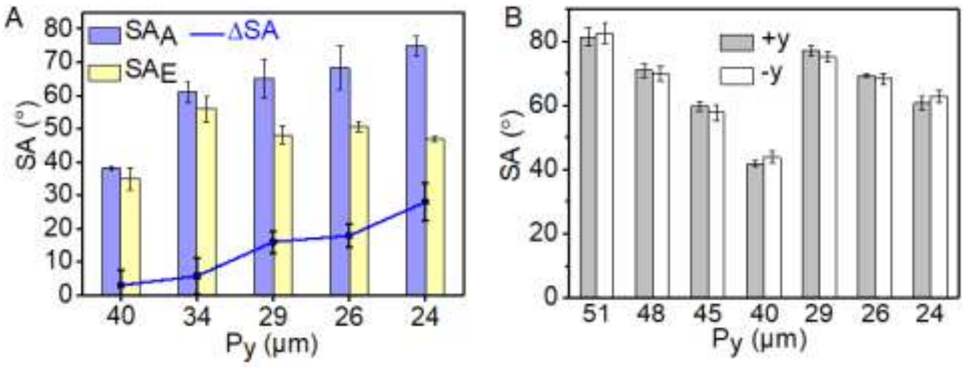

Figure S4. Sliding angle of a water droplet in x and y directions on TMAS. (A) Dependence of sliding angles in direction A (SA_A) and direction E (SA_E), and the differences of SA ($\Delta SA = |SA_A - SA_E|$) on Py. The water droplet was 6 µL. (B) Dependence of SA along y axis on Py. The water droplet was 4 µL. Each data point represents the mean value of three measurements. Standard deviations are indicated by error bars.

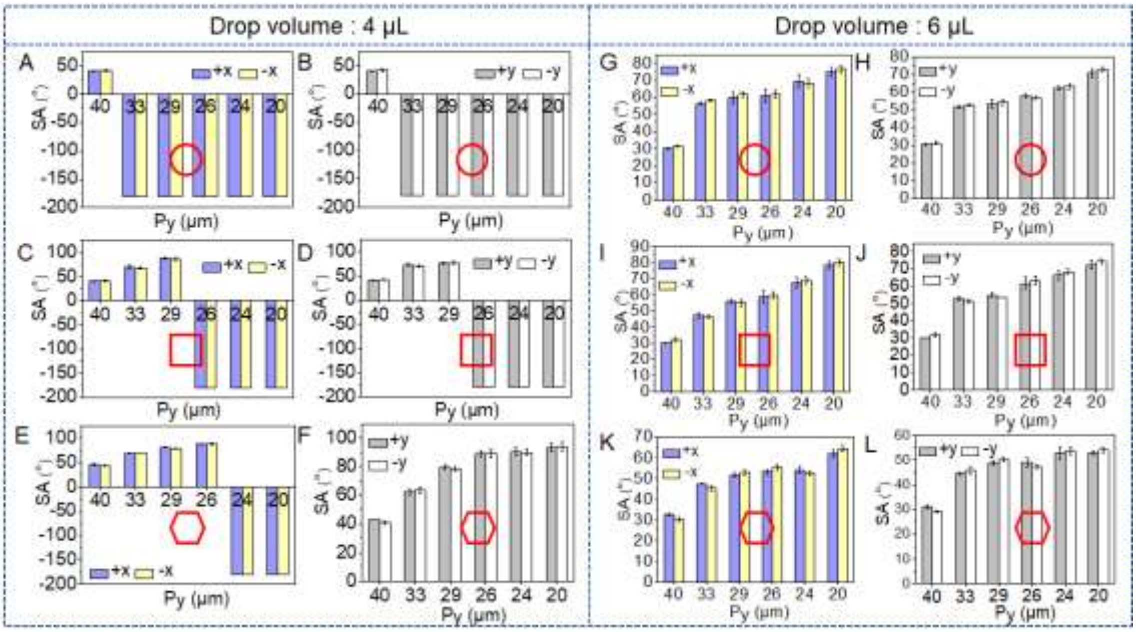

Figure S5. Sliding angle of a water droplet on the circular, squared and hexagonal micropillars arrays with different Py in x and y direction with drop volume of 4 and 6 µL. Each data point represents the mean value of three measurements. Standard deviations are indicated by error bars.

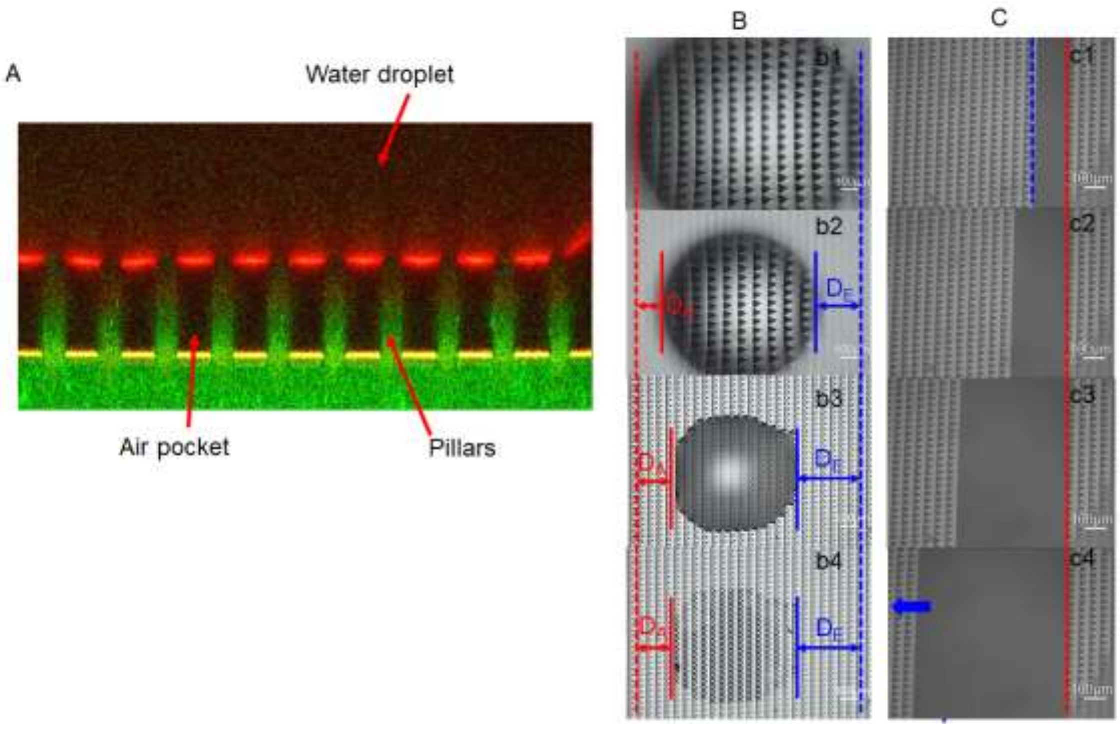

Figure S6. The state of water droplet on TMAS. (A) Fluerecence image of a 2 μL water droplet on TMAS. (B) Top-view of a 2 μL water droplet evaporating on TMAS with (b1)–(b4) corresponding to the time of 10, 429, 704 and 791 s, respectively.The moving distances of TCL in the directions of A and E are indicated by D_A and D_E, respectively. (C) Top-view images of water (4 μL) penetrating between the pillars on TMAS with (c1)–(c4) corresponding to the time of 0, 28, 55 and 78 s, respectively. It should be mentioned that the droplet was forced into the gaps among pillars.

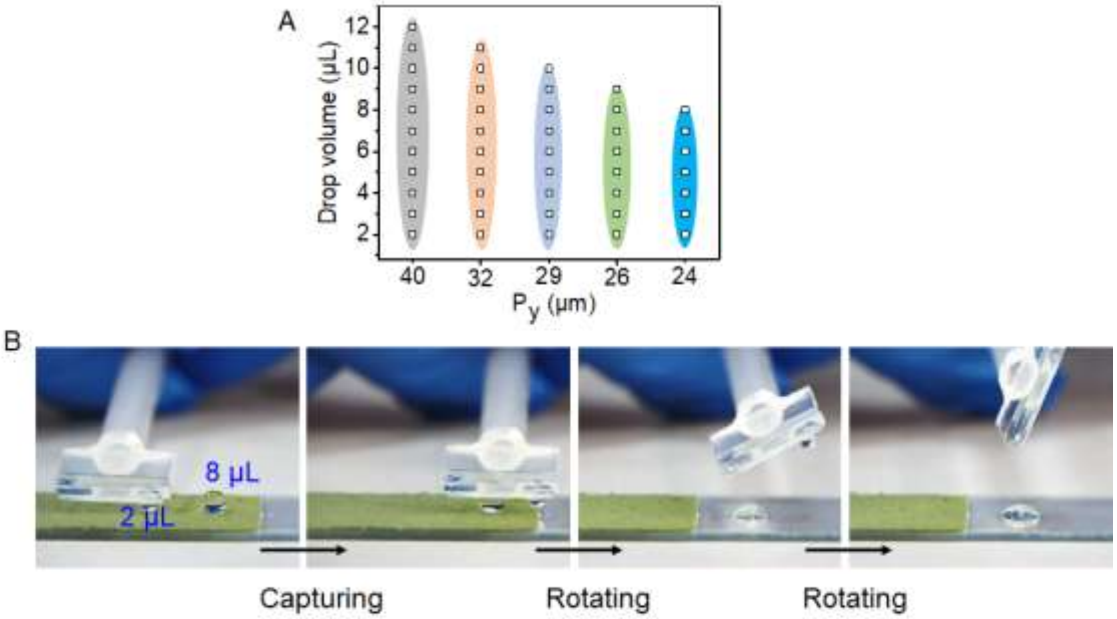

Figure S7. The ability of TMAS-y100% to transfer droplets. (A) The volume of a droplet TMAS with various Py can transfer. (B) Transferring several water droplets with various volumes at the same time. Taking two droplets of 2 and 8 μL as the example.

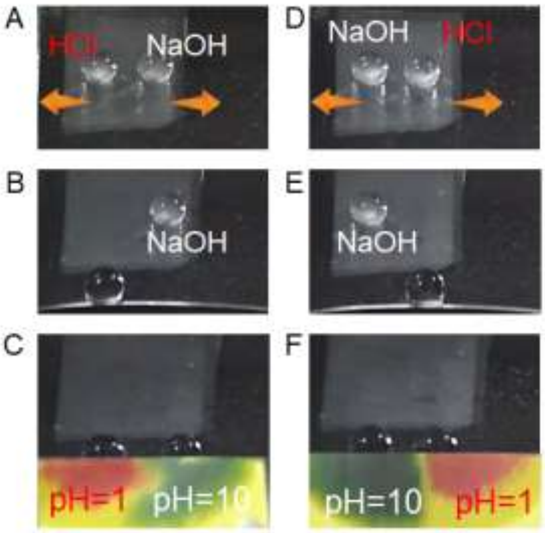

Figure S8. The ability of TMAS to recongnize acids and bases. TMAS was placed with one HCl droplet (pH = 1)and one NaOH droplet (pH = 10) of 6 μL. By slowly stretching TMAS, HCl droplet slid down first and then NaOH droplet (A-C). The position shift of the two droplets will not change the sliding sequence (D-F). The orange arrows indicate the stretching direction.

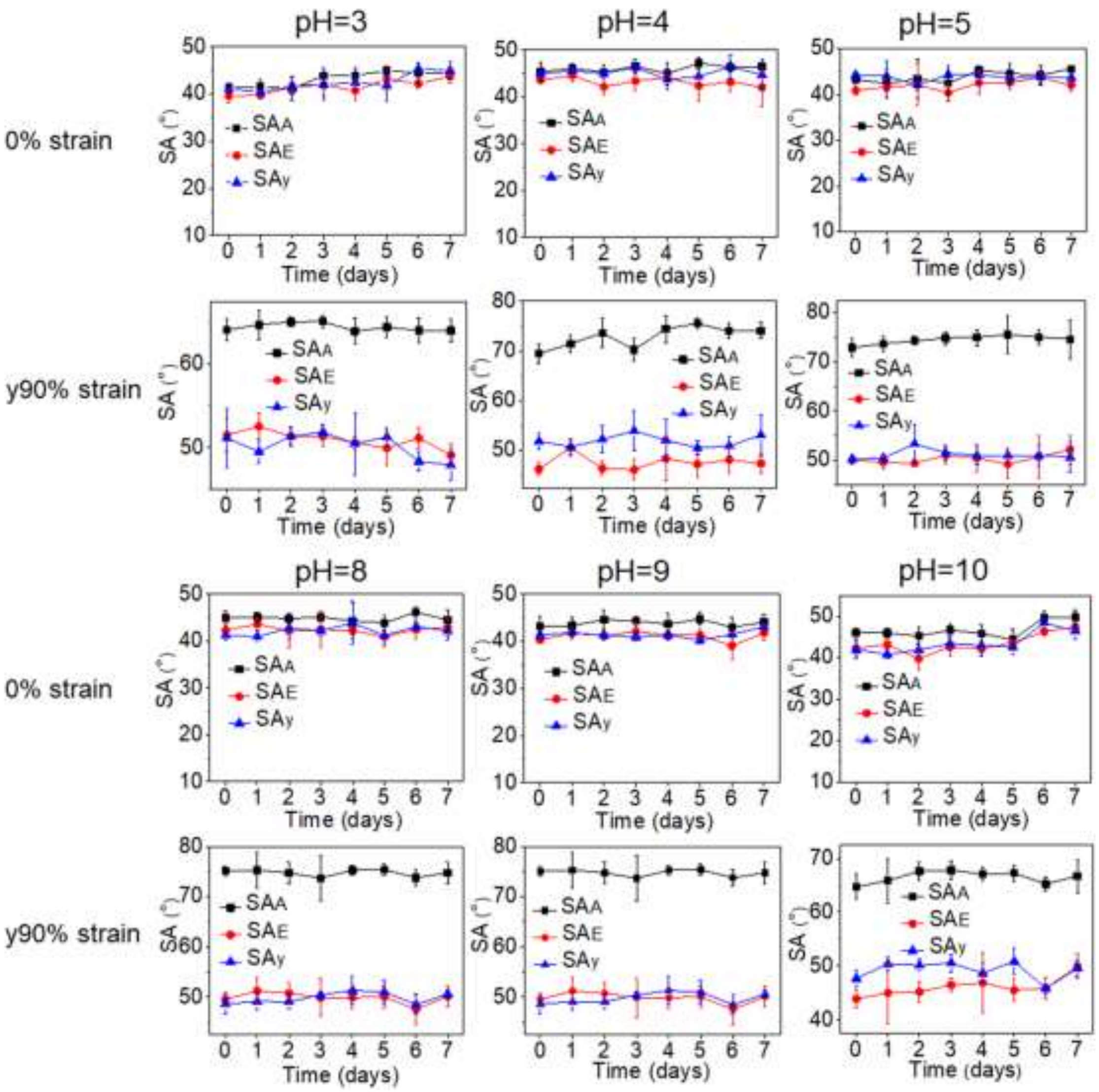

Figure S9. Sliding angle of a droplet on TMAS after the treatment in acidic or basic solution. The sliding angles in direction A (SA_A), direction E (SA_E) and direction y (SA_y) of TMAS-y0% and TMAS-y90%, which were immersed in solutions with pH values of 3, 4, 5, 8, 9 and 10 for 1-7 days. Each data point represents the mean value of three measurements. Standard deviations are indicated by error bars.

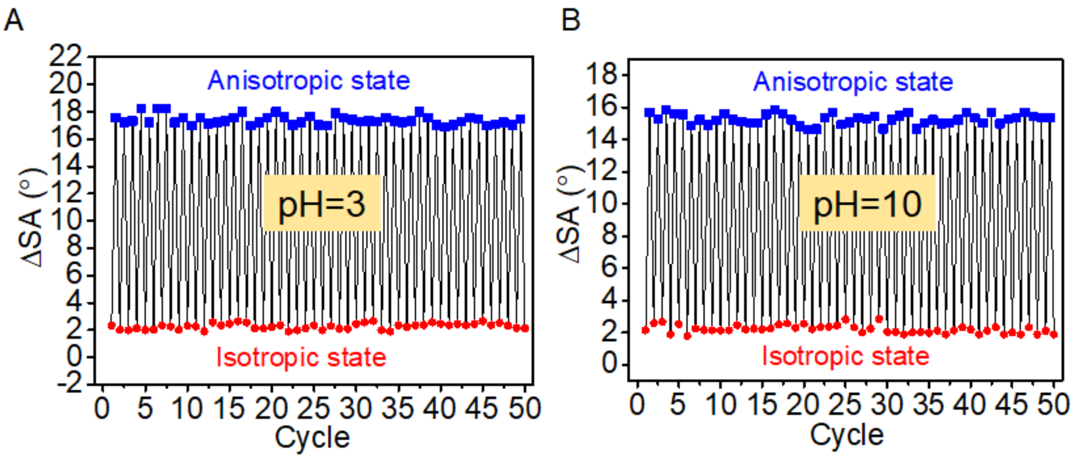

Figure S10. Cyclic switching between isotropic and anisotropic droplet adhesion by application of uniaxial dynamic strain and relaxation, showing the full reversibility. The TMAS-y90% is soaking in (A) acidic and (B) alkaline solution for 7 days, respectively.
